# No end to cholera without basic water, sanitation and hygiene

**DOI:** 10.2471/BLT.18.213678

**Published:** 2018-06-01

**Authors:** Maggie Montgomery, Megan Wilson Jones, Ibrahim Kabole, Rick Johnston, Bruce Gordon

**Affiliations:** a Water, sanitation, hygiene and health unit, World Health Organization, Geneva, Switzerland.; b WaterAid, UK, London, England.; c WaterAid, Dodoma, United Republic of Tanzania.

Safe water, sanitation and hygiene are crucial in protecting people from cholera. Improving water and sanitation services and general hygiene have proven effective in controlling and eliminating cholera in many countries. In the 47 low- and middle-income countries affected by cholera, only 79% and 44% of the population uses basic water and sanitation services respectively, compared to 94% and 79% in low- and middle-income countries without cholera.^1^

The oral cholera vaccine is perceived as an interim solution that can be deployed in advance of, or together with, investments in water sanitation and hygiene.^1^ As oral cholera vaccine is currently being considered as part of Gavi, the Vaccine Alliance’s portfolio, the vaccine’s use in endemic settings could become even more common.^2^ However, a more widespread use of oral cholera vaccine should not come at the expense of investing in and sustaining water sanitation and hygiene services, particularly in cholera hotspot areas, such as urban slums and remote rural villages that pose logistical and technological challenges.

Oral cholera vaccine comes at a cost. In Zambia each dose of vaccine costs 2.31 United States dollars (US$) and the benefits are limited to *Vibrio cholerae*, with a protective effect of five years at most. Efforts to improve water sanitation and hygiene, on the other hand, have a relatively high return: US$ 4.30 for every dollar invested in water and sanitation,^3^ in addition to prevention of most waterborne diseases and time saved from not having to fetch water. 

Furthermore, several water sanitation and hygiene interventions can be implemented quickly and cheaply, such as point-of-use water treatment and safe storage, community action to end open defecation, provision of soap and promotion of handwashing. The United Nations Children’s Fund (UNICEF) and World Health Organization (WHO) Joint Monitoring Programme reports that many low-income countries, such as Cambodia and Ethiopia, have made rapid progress on, for example, eliminating open defecation, which has been shown to significantly reduce diarrhoeal diseases.^4^^,^^5^

The reasonable alternative would be to pursue both oral cholera vaccine and water sanitation and hygiene efforts in parallel as done in, for example, Zanzibar, the United Republic of Tanzania and in Zambia.

Zanzibar, the United Republic of Tanzania, has worked with health sector and water, hygiene and sanitation partners to develop a 10-year cholera elimination plan,^6^ aligned to the new global roadmap. The plan specifically targets five hotspots where infection rates ranged between 80% and 95%. Cholera epidemics in these hotspots are closely linked to poor water sanitation and hygiene access. The plan involves high-level political leadership, ensuring engagement of relevant ministries and encouraging donors to support and invest in the plan.

In Zambia, the cholera outbreak which started in 2017 in Lusaka, now totals over 5000 cases and has resulted in nearly 100 deaths.^7^ Intensive door-to-door hygiene promotion, hygiene kits distribution and enhanced water-quality testing and monitoring in the most affected sub-districts of Lusaka is helping to curb the outbreak in these areas. However, the elimination of cholera in Zambia will require investing in both short- and long-term water sanitation and hygiene services in all hotspots.

The Global Task Force on Cholera Control considers water, sanitation and hygiene investments as the foundation to meeting the goal of reducing cholera deaths by 90% by 2030.^8^ We argue that three main actions need to be taken to ensure that such investments are prioritized as part of the renewed efforts to end cholera.

First, when countries request oral cholera vaccine, they should engage in water sanitation and hygiene efforts. These efforts should include a systematic analysis of water sanitation and hygiene needs, priorities and potential financing mechanisms. In the recent roll-out of oral cholera vaccination in Malawi, although water and sanitation conditions were taken into account in prioritizing target populations, concrete actions on water and sanitation were not mentioned.^9^ A joint vaccination and hygiene campaign along with securing political and financial commitments on water and sanitation would advance prevention and control of cholera in Malawi.

Second, efforts should be made to ensure that initiatives to strengthen health systems and provide quality care devote sufficient resources for providing and sustaining water and sanitation services, especially in cholera treatment centres. The response that WHO, UNICEF and partners are developing to address the United Nations Secretary General’s call for action on water, sanitation and hygiene in health-care facilities provides new momentum to address these inadequacies.^10^

Third, donors and partners must align behind national multisectoral cholera control plans, not simply invest in stand-alone interventions. This shift will require understanding the political dynamics and support for common metrics and accountability.

A shared vision and unanimous agreement among Member States, partners and donors to prioritize broader social and environmental determinants of health, including water, sanitation and hygiene, is needed to end cholera. A proposed World Health Assembly resolution seeks to promote this consensus, ensure effective multisectoral collaborations and address cholera in tandem with other diarrhoeal diseases.
